# Early Blood Transfusion After Kidney Transplantation Does Not Lead to dnDSA Development: The BloodIm Study

**DOI:** 10.3389/fimmu.2022.852079

**Published:** 2022-03-31

**Authors:** Thomas Jouve, Johan Noble, Hamza Naciri-Bennani, Céline Dard, Dominique Masson, Gaëlle Fiard, Paolo Malvezzi, Lionel Rostaing

**Affiliations:** ^1^ Nephrology, Hemodialysis, Apheresis and Kidney Transplantation Department, University Hospital Grenoble, Grenoble, France; ^2^ Faculty of Health, Univ. Grenoble Alpes, Grenoble, France; ^3^ Human Leukocyte Antigen (HLA) Laboratory, Etablissement Français du Sang (EFS), La Tronche, France; ^4^ Urology and Kidney Transplantation Department, University Hospital Grenoble, Grenoble, France

**Keywords:** kidney transplantation, induction treatment, blood transfusion, HLA sensitization, anti-thymocyte globulin (ATG)

## Abstract

Outcomes after kidney transplantation are largely driven by the development of *de novo* donor-specific antibodies (dnDSA), which may be triggered by blood transfusion. In this single-center study, we investigated the link between early blood transfusion and dnDSA development in a mainly anti-thymocyte globulin (ATG)-induced kidney-transplant cohort. We retrospectively included all recipients of a kidney transplant performed between 2004 and 2015, provided they had >3 months graft survival. DSA screening was evaluated with a Luminex assay (Immucor). Early blood transfusion (EBT) was defined as the transfusion of at least one red blood-cell unit over the first 3 months post-transplantation, with an exhaustive report of transfusion. Patients received either anti-thymocyte globulins (ATG) or basiliximab induction, plus tacrolimus and mycophenolic acid maintenance immunosuppression. A total of 1088 patients received a transplant between 2004 and 2015 in our center, of which 981 satisfied our inclusion criteria. EBT was required for 292 patients (29.7%). Most patients received ATG induction (86.1%); the others received basiliximab induction (13.4%). dnDSA-free graft survival (dnDSA-GS) at 1-year post-transplantation was similar between EBT+ (2.4%) and EBT- (3.0%) patients (chi-squared p=0.73). There was no significant association between EBT and dnDSA-GS (univariate Cox’s regression, HR=0.88, p=0.556). In multivariate Cox’s regression, adjusting for potential confounders (showing a univariate association with dnDSA development), early transfusion remained not associated with dnDSA-GS (HR 0.76, p=0.449). However, dnDSA-GS was associated with pretransplantation HLA sensitization (HR=2.25, p=0.004), hemoglobin >10 g/dL (HR=0.39, p=0.029) and the number of HLA mismatches (HR=1.26, p=0.05). Recipient’s age, tacrolimus and mycophenolic-acid exposures, and graft rank were not associated with dnDSA-GS. Early blood transfusion did not induce dnDSAs in our cohort of ATG-induced patients, but low hemoglobin level was associated with dnDSAs-GS. This suggests a protective effect of ATG induction therapy on preventing dnDSA development at an initial stage post-transplantation.

## Introduction

Outcomes after kidney transplantation have improved over the last 70 years. However, long-term graft survival remains limited by chronic antibody-mediated rejection (cABMR) (1, 2), treatment adherence, and toxicities related to immunosuppression (especially calcineurin Inhibitors [CNIs]) (3–6). The development of *de novo* donor-specific antibodies (dnDSA) is one of the main hurdles that limits long-term graft survival.

Prevention of dnDSA development is of utmost importance due to the lack of an efficient treatment to limit DSA antibody-related allograft injuries (7–9). Educating patients to adhere to their immunosuppressive drugs is a cornerstone for dnDSA prevention. In addition, an optimal immunosuppressive strategy must be sought.

The usual sensitizing events, before or following transplantation, are blood-product transfusions (e.g., red blood cells [RBC]), pregnancy, or any history of solid-organ transplantation. RBC transfusion is frequently given to chronic kidney-disease patients, especially early after kidney transplantation in this setting of acute renal injury and due to the potential blood loss associated with surgical procedures. Avoidance of early post-kidney transplant RBC transfusion using erythropoiesis-stimulating agents (ESAs) is a goal even though we do not really know if ESAs are very efficient in that setting.

Among the sensitizing events after kidney transplantation, early RBC transfusion was shown to induce dnDSA development (10, 11). The study by Ferrandiz et al. considered transfusion performed in the first-year post-transplantation: they found transfusion was a risk factor for DSA formation and antibody-mediated rejection11. A specific analysis of sensitization against RBC donors was performed by Hassan et al.: they reported a higher rate of DSA development in post-kidney transplantation recipients that had undergone a transfusion, regardless of the transfusion chronology (i.e., early and/or late transfusion events) (12). However, data are scarce from this setting, and the impact of sensitizing events is highly dependent on the type of immunosuppressive regimen. Low adherence and therefore low exposure to tacrolimus induces higher rates of DSAs (1, 2). The impact of the induction strategy has not been estimated in previous studies. A depleting compared to a non-depleting induction therapy may play an important role in dnDSA development (13, 14).

In this study, we investigated the effect of early (within 3 months post-transplantation) blood transfusion (EBT) on dnDSA-free kidney-allograft survival (dnDSA-GS) in a retrospective cohort of kidney recipients that received (mainly) depleting anti-thymocyte globulins (ATG) as the induction therapy.

## Patients and Methods

All patients that received a kidney between January 1st 2004 and June 30th 2015 in our university hospital were considered for inclusion in this study. Luminex ^®^ HLA antibody data were gathered from all patients: indeed, Luminex screening was initiated in 2004 in our center. A minimum graft survival of 90 days was required for inclusion to avoid early competitive risks. The threshold for anti-HLA alloantibody positivity was set at a 1000 mean fluorescence intensity (MFI), using the Immucor platform.

RBC transfusion data were obtained from the regional blood bank, which registered all transfusion events, thereby providing a full picture (every single RBC unit was delivered by the regional blood bank, ensuring no missing data). The policy for RBC transfusion was mainly based on hemoglobin levels and the presence of a heart disease: the hemoglobin threshold for transfusion was 8 g/dl in heart disease-free patients, and 10 g/dl in patients with a hearth disease. All transfused RBC were leukodepleted using a leukodepletion filter fixed on collection devices. Quality controls were randomly performed to ensure that the residual leucocyte count was below 1x10*6/RBC unit. The volume of each packed RBC unit was 230-300 mL. The RBC were not irradiated excepted for the patient with deep immunosuppression or with hematological malignancy.Hemoglobin and tacrolimus trough levels were recorded from every available blood test (at least a monthly follow-up, more frequently as clinically advised). Drug prescriptions were evaluated at M3, M6, M12, and then annually. Luminex screening for HLA antibody data was performed at the time of transplantation, at 3- and 6-months post-transplantation, then annually, and also if there was an acute kidney event, with identification whenever the screening was positive.

Regarding post-transplant immunosuppression, all the recipients received an induction therapy based either on ATG or basiliximab. Patients receiving a first kidney transplant, at low allosensitization risk and/or high infection/cancer risks, were treated with basiliximab. Maintenance immunosuppression was based on tacrolimus, mycophenolic acid (MPA), and prednisone. Trough levels of tacrolimus were between 7 and 10 ng/mL during the first 3 months post-transplantation and thereafter between 4.5–6 ng/ml. Patients were usually weaned off steroids after 3 months except in cases of IgA nephropathy or pretransplant HLA sensitization, or if the surveillance kidney-allograft biopsy (performed at month 3 post-transplantation) showed borderline changes or subclinical acute rejection. Cytomegalovirus (CMV) and Pneumocystis jirovecii prophylaxes were based on valganciclovir (900mg/d, adapted to eGFR, given for 3 to 6 months according to the CMV donor/recipient serostatus), and sulfamethoxazole-trimethoprim given at 800/160 mg every other day for 6 months (adapted to eGFR as well), respectively.

Kaplan–Meier curves and Cox’s proportional-hazard regression analyses were used to assess dnDSA-free graft survival (dnDSA-GS). We also performed a subanalysis among patients without any HLA sensitization before transplantation to assess anti-HLA antibody development, regardless of their target, and the predictors of these HLA antibodies. Covariates possibly evolving over time after transplantation were considered as time-dependent covariates in the different Cox’s models (e.g., medication dosages, tacrolimus trough levels, hemoglobin). In order to evaluate possible confusion factors, we systematically included EBT in the multivariate analysis, despite its absence of association with dnDSA development. Schoenfeld’s residuals were tested to evaluate departure from the proportionality assumption, i.e., to test the stability of the regression coefficients over time. The Wilcoxon test was used to make a comparison between two groups for continuous covariates. The chi-squared test was used to compare discrete covariates between two groups. All analyses were performed using the R statistical software.

The study was conducted according to the guidelines of the Declaration of Helsinki and approved by CNIL (French National committee for data protection; approval number 1987785v0). The biobank collection number is BRIF BB-0033-00069. The patients provided their written informed consent to participate in this study. No potentially identifiable human images or data is presented in this study.

The raw data supporting the conclusions of this article will be made available by the authors, without undue reservation.

## Results

Between 2004 and 2015, a total of 1088 kidney transplantations were performed in our center. Following exclusion criteria (i.e.: patients with a pre-transplantation DSA, or with graft survival of less than 90 days), the study cohort comprised 981 kidney-transplant recipients (49 [5%] patients lost their graft before 3 months, 59 [6%] had either a pre-transplantation DSA or no clear HLA antibody profile before transplantation). Median follow-up time was 9.07 years (IQ 6.57–12.2).

The induction therapy was based on ATG for 845 patients (86.1%) and basiliximab for 132 patients (13.4%). Information regarding induction therapy was missing for 4 patients (0.04%). Maintenance immunosuppression was based on tacrolimus, mycophenolate mofetil, and prednisone. The median trough tacrolimus level was 9.4 ng/mL (1st Q. 7.8, 3rd Q 11.3) over the first 3 months and 5.8 ng/mL (1st Q 5.3, 3rd Q 6.35) thereafter. The median dose of mycophenolate mofetil prescribed was 1 g per day. Patients were usually weaned off steroids after 3 months except in cases of IgA nephropathy, pretransplant HLA sensitization, or where a surveillance kidney-allograft biopsy performed at month 3 post-transplantation showed borderline changes or subclinical acute rejection. The median duration of steroid prescription was 175.5 (1st Q. 97, 3rd Q 643) days. A total of 155 patients (16%) remained on steroids at the time of last follow-up.

Demographic characteristics and potential confounders are detailed in **Table 1**. Patients in the early blood-transfusion (EBT+) group were more fragile: i.e., they were older, had longer cold ischemia time, and lower M3 estimated glomerular-filtration rates (eGFR) than patients in the EBT- group. There was no significant association with the initial nephropathy (chi-squared test p=0.079), but a trend toward more diabetic and hypertensive nephropathies (i.e., comorbid patients) in the EBT group. Also, early blood transfusion was more often performed in the ATG group than in the basiliximab group (31.1% in the ATG group, 20.5% in the basiliximab group, Fisher exact test p=0.014). The median hemoglobin level triggering the blood transfusion was 8.3 g/dl [1^st^ Q 7.9, 3^rd^ Q 9.7]. Overall, there were 86 [1^st^ Q 60, 3^rd^ Q 112] hemoglobin measurements available per patient over their post-transplantation follow-up, with a median value of 12.8 g/dl (standard deviation 1.77 g/dl).

**Table 1:** Comparative demographics and potential confounders between transfusion groups.

	Early RBC - (*N*=689)	Early RBC + (*N*=292)	Total (*N*=981)	*p*-value
Recipient’s age, yrs	50.02 (14.71)	55.86 (12.80)	51.76 (14.42)	<0.001
Recipient’s gender, female	243 (35.3%)	130 (44.5%)	373 (38.0%)	0.006
Graft rank	0.22 (0.48)	0.24 (0.51)	0.22 (0.49)	0.779
Time on dialysis, yrs	3.99 (4.89)	4.58 (4.42)	4.18 (4.75)	<0.001
Pretransplantation HLA sensitization	157 (22.8%)	85 (29.1%)	242 (24.7%)	0.036
Donor type				<0.001
- DCD	22 (3.2%)	8 (2.7%)	30 (3.1%)	
- BD	566 (82.1%)	276 (94.5%)	842 (85.8%)	
- LD	101 (14.7%)	8 (2.7%)	109 (11.1%)	
ATG induction, yes	582 (84.7%)	263 (90.7%)	845 (86.1%)	0.014
Cold ischemia time, min	909.76 (503.41)	1112.12 (466.43)	970.06 (501.08)	<0.001
dnDSA	85 (12.3%)	29 (9.9%)	114 (11.6%)	0.282
Nbr. of post-transplantation transfusion	0.23 (1.07)	2.94 (3.63)	1.03 (2.50)	<0.001
Nbr. of HLA mismatches	5.46 (0.93)	5.33 (0.86)	5.42 (0.91)	0.043
eGFR at M3 post-KTx, mL/min/1.73m^2^	58.34 (21.86)	52.44 (22.00)	56.62 (22.05)	<0.001
BPAR up to 1-year post-KTx.	21 (3.0%)	10 (3.4%)	31 (3.2%)	0.758
Follow-up time, yrs	9.63 (3.63)	8.48 (4.47)	9.28 (3.93)	<0.001

RBC, red blood-cell transfusion; yrs, years; HLA, human leukocyte antigen; dnDSA, de novo donor-specific alloantibody; DCD, donation after circulatory death; BD, brain dead; LD, living donor; eGFR, estimated glomerular-filtration rate; M, month; BPAR, biopsy-proven acute rejection; KTx, kidney transplantation.

The p-values are from chi-squared tests or Wilcoxon tests depending on type of data.

HLA mismatch numbers are based on a 2-digits level for A, B, DR, DQ.

Among the 981 patients, 292 (29.7%) received at least one RBC transfusion within the first 3 months post-transplantation. A total of 114 patients (11.6%) developed a *de novo* DSA (dnDSA): 19 patients (1.9%) developed a class 1 dnDSA, 83 (8.5%) a class 2 dnDSA, and 12 (1.2%) developed dnDSAs against both classes. **Table 2** describes the characteristics of the immunodominant DSAs for all targets encountered in at least two different patients.

**Table 2:** Description of immunodominant DSAs identified in the cohort.

iDSA	Min MFI	Max MFI	Number of patients	Number of sera
**DQ2**	410	19,348	14	27
**DQ7**	851	17,860	12	32
**DQ3**	1551	17,176	10	15
**DQ5**	1233	20,480	10	16
**DQ6**	319	16,436	9	26
**DR4**	1187	11,745	5	9
**A24**	659	4886	4	6
**DQ4**	1031	15,596	4	13
**DQ9**	253	1461	4	5
**A1**	964	11,232	3	3
**DQ8**	1585	10,223	3	11
**DR53**	1546	17,004	3	8
**A2**	4664	4664	2	2
**C7**	756	11,911	2	9
**DP2**	598	2755	2	3
**DQ1**	1060	11,932	2	2
**DQA3**	1362	3700	2	5
**DR1**	4169	10,590	2	5
**DR15**	3659	21,320	2	2

MFI, mean fluorescence intensity; DSA, donor-specific alloantibody; iDSA, immunodominant DSA.

Minimum and maximum values of the immunodominant DSA are shown, together with the number of patients developing the corresponding DSA. The number of different sera containing the DSA is also shown.

Over the follow-up period, there were 213 death-censored graft losses, i.e., 21.7%. As expected, there was a clear relationship between graft loss (death-censored) and dnDSA development (p<0.001). Demographic characteristics and potential confounders of patients with and without a dnDSA are detailed in **Table 3**.

**Table 3:** Description of the dnDSA^-^ and dnDSA^+^ groups.

	No dnDSA (*N*=867)	dnDSA^+^ (*N*=114)	Total (*N*=981)	*p*-value
Recipient’s age, yrs	52.19 (14.30)	48.49 (14.95)	51.76 (14.42)	0.014
Recipient’s gender, F	330 (38.1%)	43 (37.7%)	373 (38.0%)	0.943
Graft rank	0.21 (0.47)	0.34 (0.58)	0.22 (0.49)	0.004
Time on dialysis, yrs	4.02 (4.59)	5.27 (5.68)	4.18 (4.75)	0.033
Pretransplantation HLA sensitization	193 (22.3%)	49 (43.0%)	242 (24.7%)	<0.001
Donor type				0.103
- DCD	29 (3.3%)	1 (0.9%)	30 (3.1%)	
- BD	737 (85.0%)	105 (92.1%)	842 (85.8%)	
- LD	101 (11.6%)	8 (7.0%)	109 (11.1%)	
Cold ischemia time, min	962.03 (502.99)	1031.04 (484.08)	970.06 (501.08)	0.234
Nbr. of HLA mismatches	5.40 (0.89)	5.59 (1.05)	5.42 (0.91)	0.250
Early transfusion	263 (30.3%)	29 (25.4%)	292 (29.8%)	0.282
eGFR at M3 post-KTx, mL/min/1.73m^2^	56.59 (22.09)	56.86 (21.85)	56.62 (22.05)	0.929
BPAR up to 1 year post-KTx.	25 (2.9%)	6 (5.3%)	31 (3.2%)	0.172
Follow-up time, days	9.28 (3.92)	9.35 (4.08)	9.28 (3.93)	0.831

yrs, year; F, female; dnDSA, de novo donor-specific alloantibody; HLA, human leukocyte antigen; DCD, donation after circulatory death; BD, brain dead: LD, living donor; eGFR, estimated glomerular-filtration rate; M, month; KTx, kidney transplantation; BPAR, biopsy-proven acute rejection.

The p-values are from chi-squared tests or Wilcoxon tests depending on the type of data.

HLA mismatch numbers are based on a 2-digits level for A, B, DR, DQ.

### Donor-Specific Antibody Development (DSA)

The number of dnDSAs detected up to 1-year post-transplantation was not associated with EBT: 21 (3.0%) patients developed a dnDSA in the EBT- group vs. 7 (2.4%) in the EBT+ group (p=0.73). When we also took the value of median hemoglobin between 3- and 6-months post-transplantation into account (in a multivariate logistic regression model, adjusting for early transfusion and median hemoglobin over this 3-month period), the effect of early transfusion remained non-significant (OR=0.58, p=0.295), as well as the effect of median hemoglobin (OR=0.85 for each 1 g/dL increase, p=0.255).

Among the 933 patients with an available 1-year transfusion history (i.e., up to one year after transplantation), 292 patients received at least one unit of RBC with the same result: i.e., transfusion during the first-year post-transplantation did not induce a higher rate of DSA development (HR 0.89, p=0.607). Most transfusions took place within the first 3 months: only 10 patients received a transfusion between month-3 and 1-year post-transplantation.

The association between dnDSAs (over the whole follow-up period) and EBT was not significant: 85 (12.33%) patients not receiving any RBCs over the first 3 months developed a DSA compared to 29 (9.9%) patients that did receive at least one transfusion of RBCs over the first 3 months (chi-squared, p=0.33). The dnDSA onset time was 2.77 years in the EBT- group [1^st^ Q 1.02, 3^rd^ Q 5.45] VS 4.73 years in the EBT+ group [1^st^ Q 1.1, 3^rd^ Q 6.79] (Wilcoxon p=0.511). In the EBT- group, 11 patients (12.5%) developed a class 1 DSA, 66 (75%) developed a class 2 DSA and 11 (12.5%) developed both class 1 and class 2 DSAs. In the EBT+ group, 9 (33%) patients developed a class 1 DSA, 17 (63%) developed a class 2 DSA and 1 patient developed both class 1 and class 2 DSAs.

**Figure 1:**
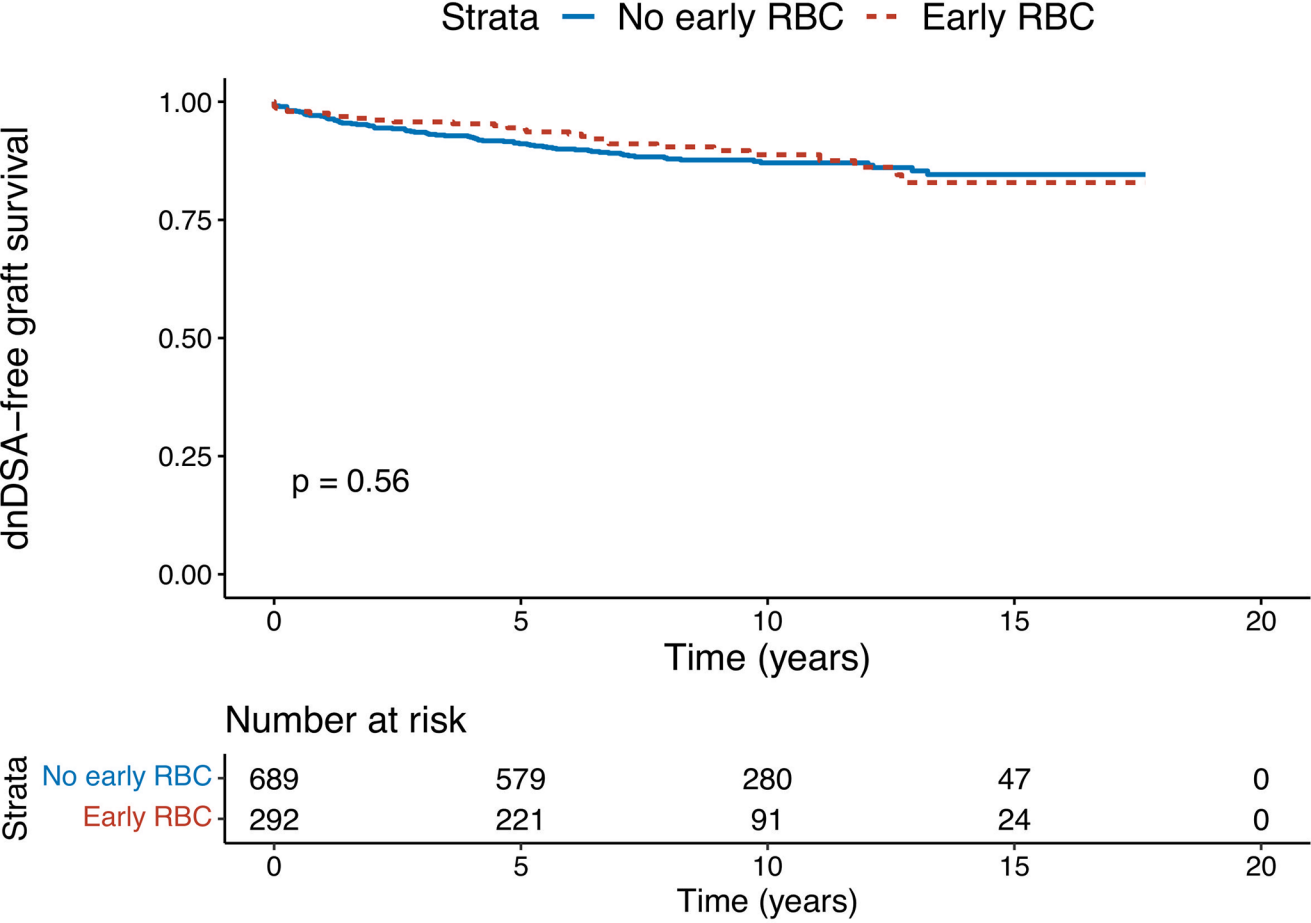
Kaplan–Meier survival analysis of early (within 3 months post-transplantation) red blood-cell transfusion on dnDSA-free graft survival. The given p-value stands for the log-rank test.

The effect of EBT on overall dnDSA-free death-censored graft survival (dnDSA-GS, i.e., up to and beyond 1 year) was not significant (HR=0.88, p=0.556). The Kaplan–Meier survival curves, depending on EBT, are shown in **Figure 1**. Significant univariate predictors (p-value threshold of 0.1) for dnDSA development were recipient’s age (HR=0.99 for each increase of 1 year, p=0.062), pre-transplantation HLA sensitization (HR=2.57, p<0.001), HLA mismatch number between donor and recipient (HR=1.27, p=0.02), tacrolimus trough levels (HR=0.88 for each increment of 1 µg/L of tacrolimus trough level, p=0.027), hemoglobin level > 10 g/dl (HR=0.33, p=0.006), graft rank (HR=1.60 for each previous kidney transplant, p=0.003), and the C/D ratio of tacrolimus (HR=1.66 for a C/D ratio <1.05, p=0.036). Non-significant predictors were recipient gender, donor type, donor age, induction strategy (ATG vs. basiliximab), MPA dose, and month-3 post-transplantation eGFR. Visual trends are provided in **Supplementary Figure 1**, showing the dnDSA-free survival curves between the 4 EBT x induction groups.

**Figure 2:**
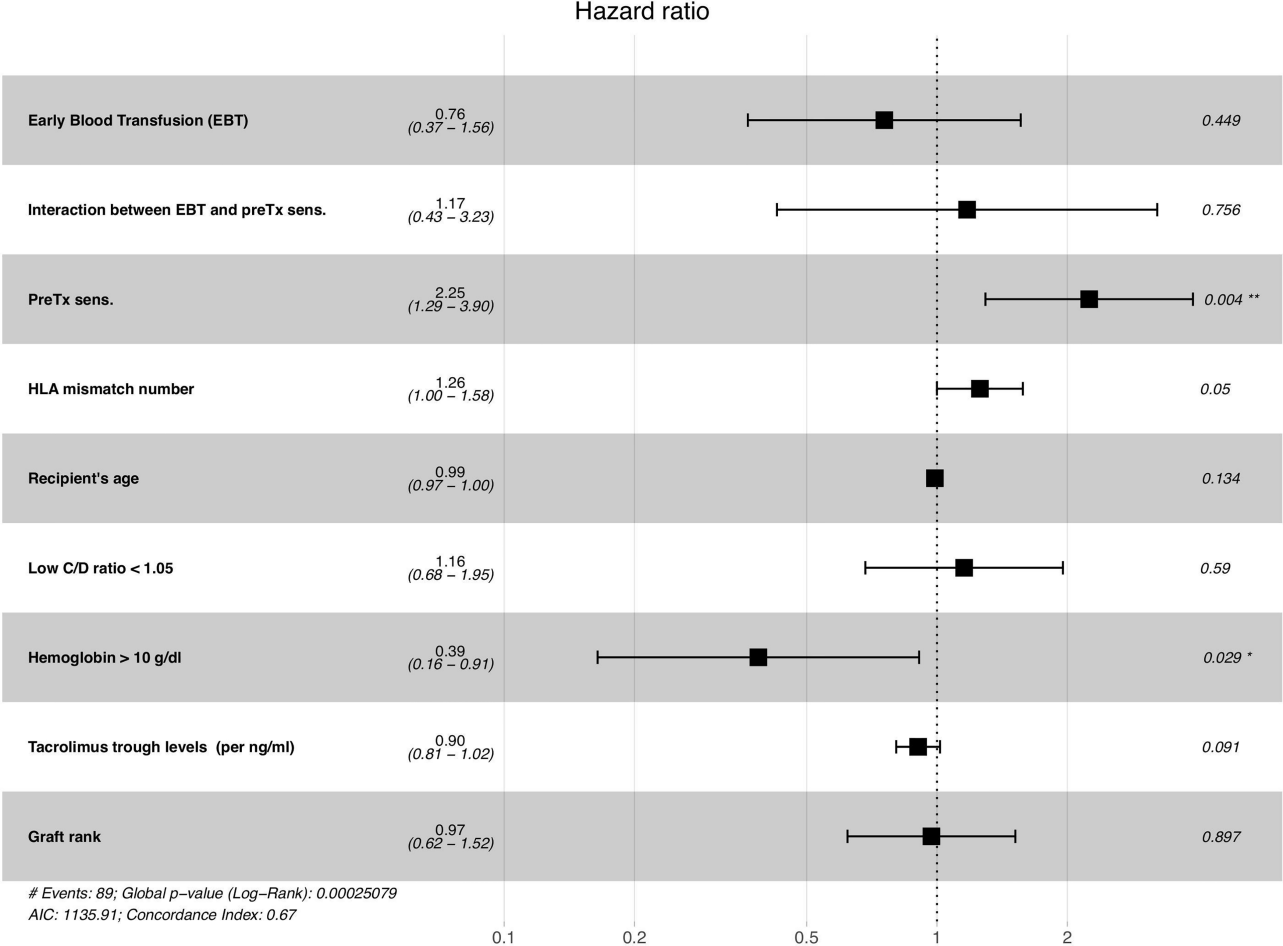
Multivariate Cox’s survival model predicting the occurrence of a dnDSA, based on significant univariate predictors of dnDSAs. PreTx = pre-transplantation; sens. = HLA sensitization (presence of anti-HLA antibody); C/D ratio = Tacrolimus concentration-to-dose ratio. Time-varying covariates were considered as such in this multivariate Cox model *p < 0.05, **p < 0.01.

In the multivariate Cox’s regression model that retained all significant univariate covariates (with a p=0.1 threshold for inclusion), EBT was not associated with dnDSA-GS (HR=0.76, p=0.449). Pre-transplantation HLA sensitization was clearly associated with dnDSA-GS (HR=2.25, p=0.004) as well as hemoglobin levels (HR=0.39 for hemoglobin of >10 g/dL, p=0.029) and the number of HLA mismatches (HR=1.26 per HLA mismatch, p=0.05). However, recipient’s age, a low C/D ratio (<1.05), tacrolimus dose, MPA dose, and graft rank were no longer significant, as represented in **Figure 2**. The interaction between early transfusion and pre-transplantation HLA sensitization was not significant either, showing no synergistic effect of EBT and pretransplant HLA sensitization. **Table 4** summarizes the DSA-free survival analyses. The effect of hemoglobin did not change with time at post-transplantation in our model: the influence of hemoglobin on dnDSA-free survival was stable over time (Schoenfeld residual test, p=0.92). On the other hand, the effect of EBT changed over time (Schoenfeld residual test, p=0.026), from protective to deleterious at around 5 years post-transplantation. This suggests that factors associated with the risk of transfusion are later associated with the risk of dnDSA.

**Table 4:** Survival analysis for DSA: univariate and multivariate models, hazard ratios (*p*-values).

	HSA		DSA	
*Covariate*	*Univariate*	*Multivariate*	*Univariate*	*Multivariate*
Pretransplantation HLA sensitization	NA	NA	2.57 (p<0.001)	2.25 (p=0.004)
Early transfusion (ref=no)	1.25 (p=0.188)	1.27 (p=0.259)	0.88 (p=0.556)	0.76 (p=0.449)
Interaction between pretr. HLA sensitization and EBT	NA	NA	NA	1.17 (p=0.756)
Number of HLA mismatches (per each HLA MM)	1.19 (p=0.046)	1.13 (p=0.247)	1.27 (p=0.02)	1.26 (p=0.050)
ATG induction (ref=basiliximab)	0.95 (p=0.822)	–	1.24 (p=0.455)	–
Age (year)	0.99 (p=0.06)	0.98 (p=0.013)	0.99 (p=0.062)	0.99 (p=0.134)
Gender (ref=F)	0.72 (p=0.037)	0.93 (p=0.706)	1.03 (p=0.866)	–
Donor age > median donor age (52 years)	0.88 (p=0.398)	–	1.07 (p=0.734)	–
Month-3 GFR < 30 ml/min/1.73m^2^	0.82 (p=0.559)	–	0.97 (p=0.933)	–
Donor type (ref=DCD, VS LV)	0.78 (p=0.323)	–	0.58 (p=0.137)	–
Tacrolimus C/D ratio (ref>1.05)	1.35 (p=0.216)	–	1.66 (p=0.036)	1.16 (p=0.590)
Tacrolimus trough level (per 1 µg/L)	1.06 (p=0.155)	–	0.88 (p=0.027)	0.90 (p=0.091)
Hemoglobin (>10g/dl)	0.43 (p=0.056)	0.41 (p=0.045)	0.33 (p=0.006)	0.39 (p=0.029)
MPA dose (per 1 g increase)	1.25 (p=0.307)	–	1.22 (p=0.464)	–
Graft rank (for each previous transplant)	3.06 (p<0.001)	2.13 (p=0.006)	1.60 (p=0.003)	0.97 (p=0.897)

DSA, donor-specific alloantibody; HSA, HLA-specific antibody; NA, not applicable; ATG, antithymocyte globulins; HLA, human leukocyte antigen; MM, mismatch; yrs, years; F, female; eGFR, estimated glomerular-filtration rate; DCD, donation after circulatory death; LD, living donor; MPA, mycophenolic acid.

To further investigate this hypothesis, we investigated the effect of late (> 1-year post-transplantation) transfusion events on dnDSA-free survival. There were only 37 patients receiving a late transfusion in our cohort, among which only 5 developed a dnDSA. Adjusting a Cox model with both EBT and late transfusion as predictors, we find non-significant yet opposite effects of EBT and late transfusion, in terms of HR (HR for EBT 0.81, p=0.36; HR for late transfusion 1.59, p=0.131). This also suggests a detrimental effect of late transfusion VS EBT.

### HLA Antibody Development (HSA)

We also focused on HLA antibodies, regardless of the HLA target, referred to as HLA-specific antibodies (HSA). For this analysis, we focused on the cohort of 739 patients without any pretransplantation HLA sensitization. The raw association between EBT and overall development of HSA (regardless of the time post-transplantation) was not significant: 115 (21.6%) in the EBT- group developed HSA, whereas 50 (24.2%) patients in the EBT+ group developed HSA (chi-square, p=0.519).

Early transfusion was not a significant predictor of HSA-free death-censored graft survival either (HR=1.25, p=0.188). Factors univariately associated with HSA-free death-censored graft survival were recipient’s age (protective, HR=0.99 for each increase of 1 year, p=0.06), male gender (protective, HR=0.72, p=0.04), the number of serologic donor/recipient HLA mismatches (HR=1.19, p=0.046), hemoglobin level > 10g/dl (protective, HR=0.43, p=0.056), and graft rank (HR=3.06 for each previous kidney transplant, p<0.001). ATG induction, month-3 eGFR, donor type and donor age, tacrolimus or MPA doses were not significantly associated with HSA-free death-censored graft survival. **Table 4** summarizes the HSA-free survival analysis. There again, visual trends are provided in **Supplementary Figure 2**, showing the dnDSA-free survival curves between the 4 EBT x induction groups.

In the multivariate analysis, adjusting for EBT and all previously mentioned significant factors, only recipient’s age (HR=0.98, p=0.013), hemoglobin level (HR=0.41, p=0.044) and graft rank (HR=2.13 per each previous transplant, p=0.006 were associated with dhHSA graft survival. Both age and hemoglobin were protective (increased age or increased hemoglobin were associated with better dnHSA-free allograft survival). These results are detailed in **Table 4**.

## Discussion

Development of a *de novo* DSA is a major threat for kidney-transplant recipients. Early post-transplant RBC transfusions, a potentially sensitizing procedure, are a potential trigger for dnDSA formation. In this study, we show that kidney transplant recipients receiving RBC transfusions within the first three months post-transplantation are not at higher risk of dnDSA formation.

Our data suggest that anemia < 10 g/dl is a risk factor for dnDSA, while early transfusion events (up to 3 months and up to 1 year) are not associated with dnDSA development. Since our maintenance immunosuppressive regimen does not change over time (except for tacrolimus exposure, which is not a significant multivariate predictor of dnDSA development), we suggest that ATG induction creates a time window of at least 3-months post-transplantation, within which transfusion is not deleterious in terms of HLA sensitization. While we adjusted our analysis on covariates that were different between ATG-treated patients and basiliximab-treated patients (recipient’s age, gender and donor type), it should be noted that a confusion factor cannot be ruled out since there was a clear indication bias for the induction therapy.

The detrimental effect of hemoglobin levels, in terms of dnDSA, can be interpreted in at least two ways. A low hemoglobin level might be associated with a higher rate of RBC transfusions, leading to more DSA, at least beyond the protection of ATG; or hemoglobin levels are a surrogate of some allosensitization risk, e.g., anemic patients undergo more inflammatory events, leading to more allosensitization.

In the study by Ferrandiz et al. (11), early (within 1-year post-transplantation) RBC transfusion was associated with an increased rate of dnDSAs, as well as more antibody-mediated rejections. However, in their study, patients mainly received basiliximab as the induction therapy. The differences observed in head-to-head comparison of 1-year dnDSA-free survival between the cohort of Ferrandiz et al. and our cohort can be explained by the different induction strategies, the rest of the immunosuppressive strategy being very similar. We hypothesize that ATG induction prevented the potentially sensitizing effect of early post-transplant blood transfusions. While the effect of induction was not a significant predictor in our analysis, the small proportion of basiliximab-induced patients leads to a low power to detect an effect of induction.

In the study by Hassan et al. (12), sensitization against a shared HLA antigen between a RBC donor and the kidney transplant was detrimental: there were more DSAs and worse overall kidney-transplant survival. In their study, ~60% of RBC transfusions were performed in the first-year post-transplantation and 40% of transfusions were performed beyond 1 year. Induction drugs used were mostly depleting. However, late RBC transfusions (i.e., >1-year post-transplantation) may impact on DSA development beyond the protective effect of a depleting induction. A more rigorous analysis would demand considering transfusion events as a time-varying covariate to decipher the intricate effects of the type of induction therapy and RBC transfusion. Indeed, the former may prevent the detrimental HLA sensitizing effect of the latter. In our analysis, we considered hemoglobin as a time-dependent covariate, and show that a low hemoglobin < 10 g/dl is indeed a risk factor for dnDSA development.

This role of ATG induction is all the more important because a RBC transfusion is frequently required after transplantation. In our center, despite the early and frequent use of erythropoiesis-stimulating agents, more than a quarter of our patients require an early RBC transfusion. It is therefore important to evaluate the impact of such RBC transfusions: different immunosuppressive regimens may prevent DSA development even when an early RBC transfusion is required.

The major limitation of our study is that it is retrospective. However, its major strengths are that i) it is very homogeneous, i.e., ATG induction, and tacrolimus plus mycophenolic acid were used as the maintenance therapy, ii) we precisely and timely monitored for the presence of anti-HLA alloantibodies using Luminex at pre- and post-transplantation, and iii) we had an exhaustive record of transfusion occurences (however, without blood donor HLA typing). Our focus on pre-transplantation DSA-free patients precludes any conclusion on the post-transfusion rebound of pre-existing DSAs. This specific investigation is not possible in our cohort of mostly pre-transplantation DSA-free patients (since we did not have a desensitization program until 2017). However, it would be very interesting to focus on these specific pre-existing DSAs, as their evolution over time post-transplantation might be different from that of dnDSAs.

## Conclusion

Our results suggest that a RBC transfusion may be safely given to kidney-transplant recipients in the early post-transplantation period (first 3 months) provided that they receive an ATG-based induction therapy. This result, although apparently at variance with previous studies, reinforces the importance of considering the type of induction therapy used when evaluating allosensitization.

## Data Availability Statement

The raw data supporting the conclusions of this article will be made available by the authors, without undue reservation.

## Ethics Statement

The studies involving human participants were reviewed and approved by CNIL (French National committee for data protection) - approval number 1987785v0. The patients/participants provided their written informed consent to participate in this study.

## Author Contributions

TJ designed the study, collected the data, performed the statistical analysis and wrote the manuscript. DM and CD checked the blood transfusion data at the blood bank and performed assessment of dn DSA tests. GF is the leader of the kidney transplant surgery team. TJ, JN, HN, and PM took care of patients within the immediate post-transplant period. Finally, LR designed the study, supervised the manuscript and edited it. All authors contributed to the article and approved the submitted version.

## Conflict of Interest

The authors declare that the research was conducted in the absence of any commercial or financial relationships that could be construed as a potential conflict of interest.

## Publisher’s Note

All claims expressed in this article are solely those of the authors and do not necessarily represent those of their affiliated organizations, or those of the publisher, the editors and the reviewers. Any product that may be evaluated in this article, or claim that may be made by its manufacturer, is not guaranteed or endorsed by the publisher.

## Supplementary Material

Supplementary Figure 1 Comparison of dnDSA-free survival curves among the 4 groups defined by EBT status (EBT- VS EBT+) and the induction strategy (Basiliximab [BAS] VS anti-thymocyte globulin [ATG]). dnDSA, *de novo* Donor Specific Antibodies; EBT, Early Blood Transfusion (within 3 months post-transplantation).

Supplementary Figure 2 Comparison of dnHSA-free survival curves among the 4 groups defined by EBT status (EBT- VS EBT+) and the induction strategy (Basiliximab [BAS] VS anti-thymocyte globulin [ATG]). dnHSA, *de novo* HLA Specific Antibodies; EBT, Early Blood Transfusion (within 3 months post-transplantation).
