# Supplementary figures and images for: Early Blood Transfusion After Kidney Transplantation Does Not Lead to dnDSA Development: The BloodIm Study
Source: Front Immunol. 2022 Mar 31;13:852079. doi: 10.3389/fimmu.2022.852079 (PMC9009267; doi:10.3389/fimmu.2022.852079)

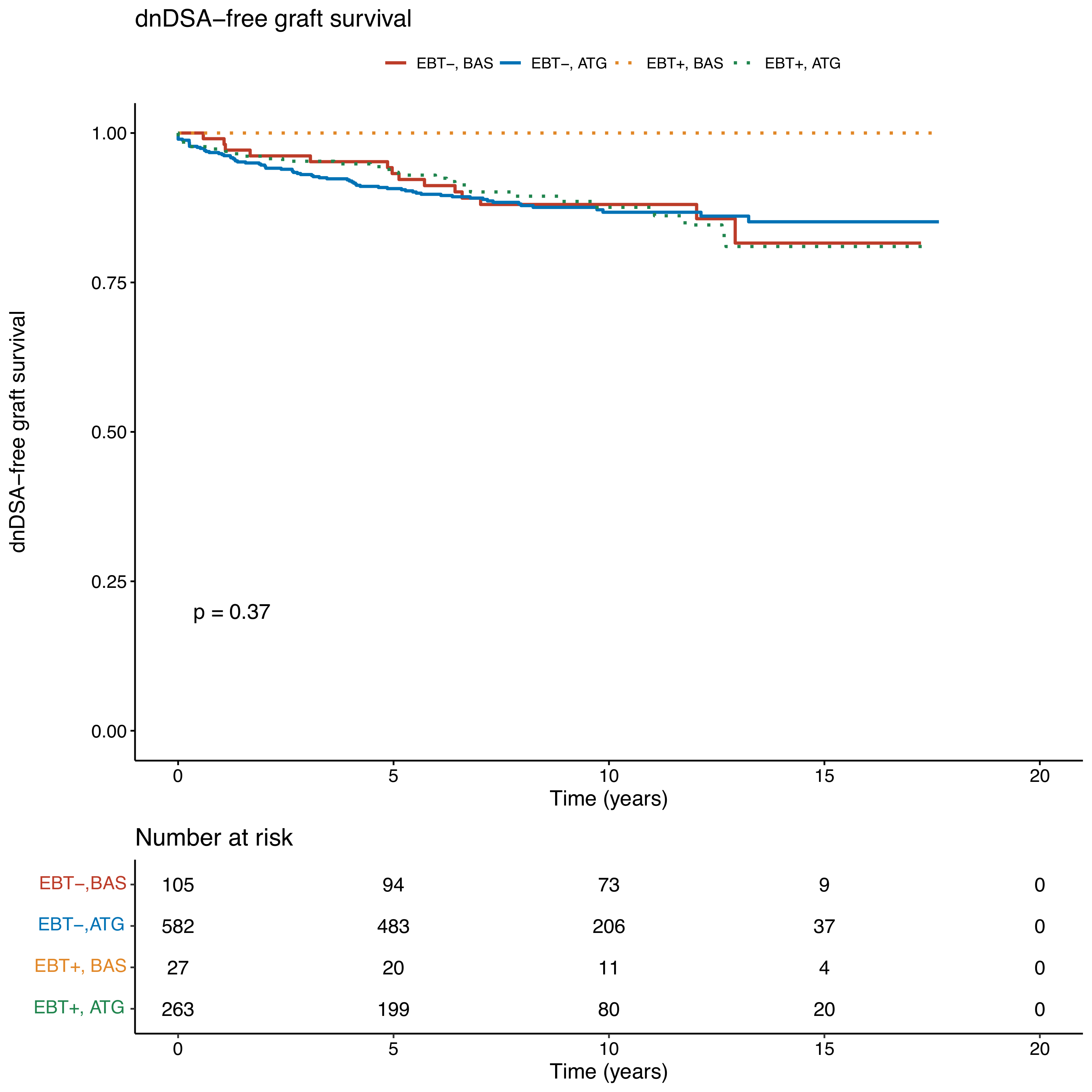

Supplement: Supplementary Figure 1 — Comparison of dnDSA-free survival curves among the 4 groups defined by EBT status (EBT- VS EBT+) and the induction strategy (Basiliximab [BAS] VS anti-thymocyte globulin [ATG]). dnDSA, de novo Donor Specific Antibodies; EBT, Early Blood Transfusion (within 3 months post-transplantation). [file Image_1.tiff]

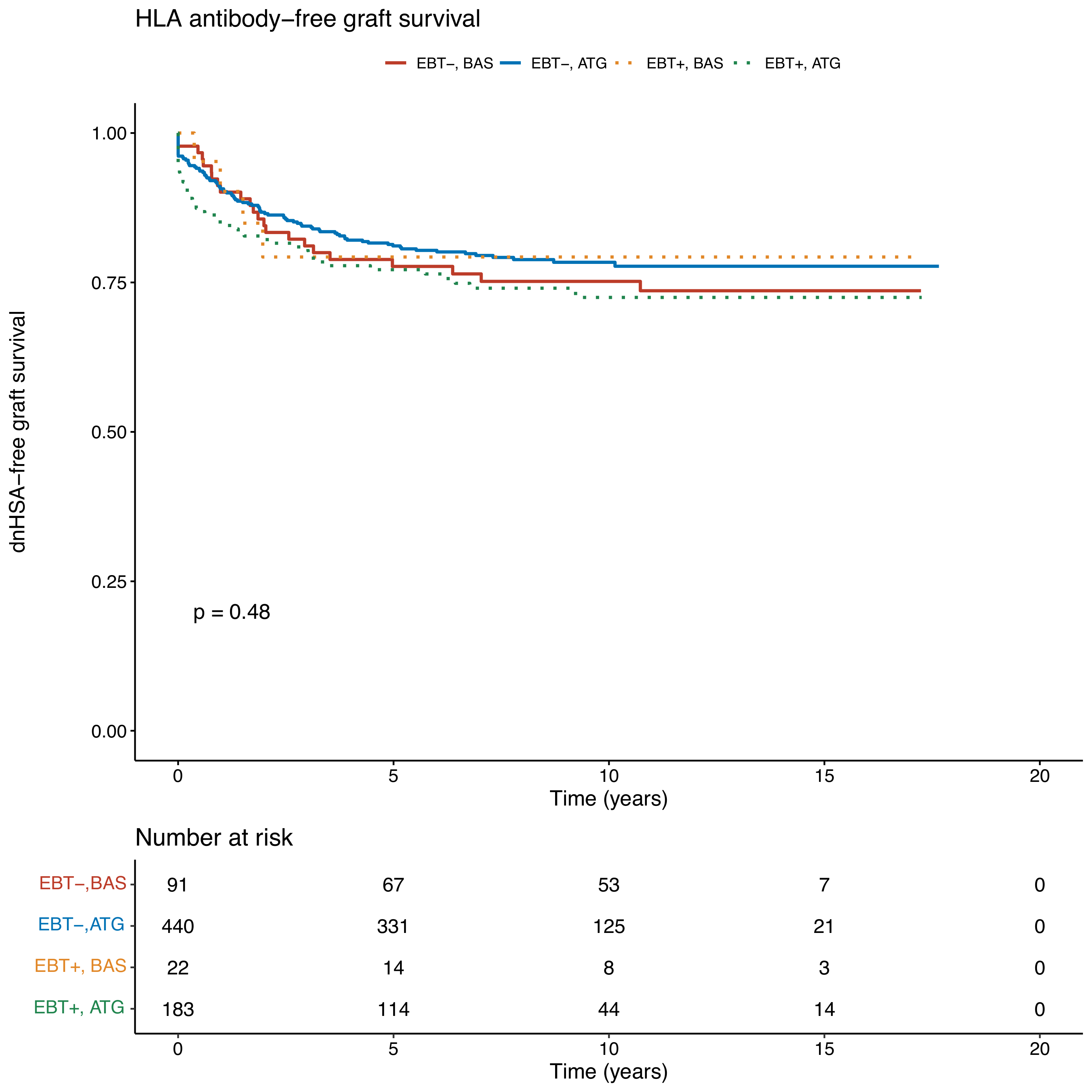

Supplement: Supplementary Figure 2 — Comparison of dnHSA-free survival curves among the 4 groups defined by EBT status (EBT- VS EBT+) and the induction strategy (Basiliximab [BAS] VS anti-thymocyte globulin [ATG]). dnHSA, de novo HLA Specific Antibodies; EBT, Early Blood Transfusion (within 3 months post-transplantation). [file Image_2.tiff]
